# Expression and clinical significance of extracellular matrix protein 1 and vascular endothelial growth factor-C in lymphatic metastasis of human breast cancer

**DOI:** 10.1186/1471-2407-12-47

**Published:** 2012-01-27

**Authors:** Qiu-Wan Wu, Hong-Qiang She, Jing Liang, Yu-Fan Huang, Qing-Mo Yang, Qiao-Lu Yang, Zhi-Ming Zhang

**Affiliations:** 1 Fujian Medical University, Fuzhou 350108, China; 2 Department of Breast Surgery, The First Affiliated Hospital of Xiamen University, Xiamen 361003, China

**Keywords:** Lymphangiogenesis, Breast cancer, Extracellular matrix protein 1, Vascular endothelial growth factor-C

## Abstract

**Background:**

Extracellular matrix protein 1 (ECM1) and vascular endothelial growth factor-C (VEGF-C) are secretory glycoproteins that are associated with lymphangiogenesis; these proteins could, therefore, play important roles in the lymphatic dissemination of tumors. However, very little is known about their potential roles in lymphangiogenesis. The aim of this study was to investigate whether correlations exist between ECM1 and VEGF-C in human breast cancer, lymphangiogenesis, and the clinicopathological characteristics of the disease.

**Methods:**

*ECM1 *and *VEGF-C *mRNA and protein expression levels in 41 patients were investigated using real-time reverse transcriptase polymerase chain reaction (RT-PCR), or immunohistochemical (IHC) staining of breast cancer tissue, matched noncancerous breast epithelial tissues, and suspicious metastatic axillary lymph nodes. D2-40 labelled lymph vessels and lymphatic microvessel density (LMVD) were counted. Correlations between *ECM1 *or *VEGF-C *protein expression levels, LMVD, and clinicopathological parameters were statistically tested.

**Results:**

The rate of ECM1 positive staining in breast cancer tissues was higher (31/41, 75.6%) than that in the corresponding epithelial tissues (4/41, 9.8%, *P *< 0.001) and lymph nodes (13/41, 31.7%, *P *< 0.001). Similarly, the VEGF-C expression rate in cancer specimens was higher (33/41, 80.5%) than in epithelial tissues (19/41, 46.3%, *P *< 0.01) or lymph nodes (15/41, 36.6%, *P *< 0.01). Higher *ECM1 *and *VEGF-C *mRNA expression levels were also detected in the tumor tissues, compared to the non-cancerous tissue types or lymph nodes (*P *< 0.05). ECM1 protein expression was positively correlated with the estrogen receptor status (*P *< 0.05) and LMVD (*P *< 0.05). LMVD in the ECM1- and VEGF-C-positive tumor specimens was higher than that in the tissue types with negative staining (*P *< 0.05).

**Conclusions:**

Both *ECM1 *and *VEGF-C *were overexpressed in breast cancer tissue samples. ECM1 expression was positively correlated with estrogen responsiveness and the metastatic properties of breast cancer. We conclude, therefore, that ECM1 and VEGF-C may have a synergistic effect on lymphangiogenesis to facilitate lymphatic metastasis of breast cancer.

## Background

Breast cancer is one of the most common cancers and the leading cause of death attributed to cancer among women in economically developed countries [1]. Unlike the hematologic system, the lymphatic system is the primary pathway to metastatic disease [2]. Recent studies have demonstrated that there is expansion of lymphatic networks within lymph nodes prior to the onset of metastasis [3]. Thus, the status of lymph node metastasis can be predicted by the presence of early lymphatic invasion. Lymphatic microvessel density (LMVD) reflects the status of lymphangiogenesis and lymphatic vessel remodeling. Increased numbers of lymphatic microvessels provide more opportunities for tumor cells to disseminate to the lymphatic system; hence, LMVD has been shown to be correlated with lymphangiogenic factors, the presence of lymphatic metastasis and a poor prognosis in breast cancer [4].

Extracellular matrix protein 1 (ECM1) was originally derived from the osteogenic mouse stromal cell line MN7 [5]. The 85-kDa glycoprotein has a close association with vascularity. It has recently been suggested that a homozygous frameshift mutation in *ECM1 *led to a failure of human mucocutaneous lymphangiogenesis [6], thus indicating a possible role for *ECM1 *in lymphangiogenesis. ECM1 is overexpressed in various malignant epithelial tumors [7-10], and has been identified as a marker of poor clinical prognosis [7-9]. However, very little is known about possible correlations between ECM1 and malignant lymphangiogenesis or lymphatic metastasis.

Lymphangiogenesis is also correlated with vascular endothelial growth factor-C (VEGF-C) expression, as demonstrated by numerous investigations [11,12]. VEGF-C initiates activation and phosphorylation of VEGFR-3 (Flt-4) which leads to PI3K-dependent Akt activation and PKC-dependent activation of the p42/p44 MAPK pathway. This process can protect lymphatic endothelial cells from apoptosis and stimulate their proliferation and migration in vitro [13,14]. In addition, genetically engineered mice conditionally overexpressing *VEGF-C *showed hyperplasia of lymphatic vessels, whereas VEGF-C-null mouse embryos completely lacked lymphatic vasculature [15]. Data from two preclinical studies provide direct evidence that an increased level of VEGF-C can promote intratumoral and peritumoral lymphangiogenesis, as well as lymphatic tumor spread to regional nodes [16,17].

The present study was designed to investigate the expression pattern of *ECM1 *and *VEGF-C *in tumor specimens, their peritumoral normal counterparts and axillary lymph nodes from 41 breast cancer patients. We also evaluated whether *ECM1 *and *VEGF-C *expression correlated with lymphatic microvessel density or the clinicopathological characteristics of the disease.

## Methods

### Patients and specimens

Fresh surgical specimens from 41 randomly selected female patients who had undergone surgery in the breast surgery department at the First Affiliated Hospital of Xiamen University (February 2009 to February 2010) were used. The average age at time of diagnosis was 53 years (range: 29-76 years). None of the patients had received preoperative treatment, such as radiotherapy or chemotherapy. Metastatic tumors from other tissue origins were excluded from the study. All cases had three samples collected as follows: breast cancer tissue, the corresponding noncancerous breast epithelial tissue (located more than 5 cm away from the tumor margins), and one of the suspicious metastatic lymph nodes from the same side of the armpit as judged by the naked eye. The specimens were divided into two parts: one part was quickly frozen in liquid nitrogen for RNA extraction, the other fixed for immunostaining and routine histological characterization.

Institutional Ethics Committee approval for the project was provided prior to commencement of the study and was in compliance with the Helsinki Declaration. Approval to conduct this study was obtained from the Human Subjects Office of the Institutional Research Board at the Xiamen University. Written informed consent was obtained from patients or their relatives. Cases were evaluated for histological type, tumor grade and histological grade (according to the Nottingham histological score) [18]. The status of lymph node metastasis, estrogen receptor (ER), progesterone receptor (PR), and human epidermal growth factor receptor 2 (HER2/neu) score were evaluated according to the American Joint Committee on Cancer (AJCC, seventh).

### Real-time RT-PCR

Total RNA was extracted using Trizol reagent according to the manufacturer's protocol (Invitrogen, USA). Reverse transcription of total RNA into cDNA was conducted using TaKaRa Reverse Transcription Reagents (Takara Bio Inc., Japan) at 37°C for 15 min, followed by 85°C for 5 s. Primers were designed using Primer Premier 5.0 software (Premier, Canada) and synthesized by Invitrogen. *ECM1 *mRNA sequence-specific primers [GenBank Accession No. NM 004425.3] with the following sequences were used: Forward (F): 5'-CAAATCTGCCTTCCTAACCG-3'; Reverse (R): 5'-AAGCAGGAGAACCGAGCC-3'. *VEGF-C *mRNA [GenBank Accession No. NM 005429.2] primers were as follows: F: 5'-GGGAAGGAGTTTGGAGT-3'; R: 5'-GCATCGGCAGGAAGT-3'. The house-keeping gene *GAPDH *mRNA was used as a reference because it is expressed at a constant level in cells. The *GAPDH *mRNA primers used were as follows: F: 5'-GAAGGTGAAGGTCGGAGTC-3'; R: 5'-GAAGATGGTGATGGGATTTC-3'. Real-time quantitative PCR was performed using the TaKaRa SYBRR^® ^Premix Ex Taq™ II PCR kit (Takara Bio Inc., Japan) in a Roche Lightcycler 480 instrument (Roche, Switzerland). Reactions were performed in 10 μl volumes with denaturation at 95°C for 5 s, annealing at 58°C for 15 s, and extension at 72°C for 20 s, over 40 cycles. The system automatically monitors binding of a fluorescent dye to double-strand DNA by real-time detection of the fluorescence emitted during each cycle of PCR amplification. Melting curves were analysed to ensure only single amplicons of the expected size were quantified. To determine the fold change in expression and to normalize *ECM1 *and *VEGF-C *expression levels, triplicates of the cycle threshold (Ct) for the target gene were averaged and divided by the average of the triplicate obtained from *GAPDH *in the same specimen.

### IHC staining and evaluation

4-μm sections of formalin-fixed, paraffin-embedded tissues were deparaffinized, stepwise rehydrated and the endogenous peroxide blocked. For both ECM1 and D2-40 staining, slides were processed with antigen retrieval which was achieved by boiling the slides in citrate buffer (pH 6.0) for 1.5 min. For VEGF-C staining, slides were boiled in an EDTA solution for 20 min before cooling. Nonspecific binding was blocked using 10% non-immune goat serum (Santa Cruz, USA.) for 10 min. Sections were incubated for 120 min at room temperature with anti-ECM1 antibody (Abcam, UK, clone SC-05) at a 1: 50 dilution, or with anti-VEGF-C antibody (Abcam, UK) at a 1: 200 dilution, or with D2-40 antibody (Abcam, UK, clone D2-40) at a 1:40 dilution. After rinsing, sections were incubated with the EnVision™ Detection System (Dako, Denmark), counterstained with haematoxylin, dehydrated, and mounted. Negative controls were processed using the same procedure, except that 10% non-immune mouse serum (Santa Cruz) was used in place of the primary antibody. No detectable staining was observed in any of the negative control slides.

LMVD was assessed by counting the number of D2-40 immunostained vessels on tissue sections. Morphometric analyses were estimated independently by two observers that had no prior knowledge of the patients' clinicopathologic data. As previous reported [19], we first identified the area containing the most stained vessels ("hot spots") by scanning the sections at low magnification (40×); then counted the number of positive vessels in two high magnification fields (200×). We defined those vessels as lymphatics if they were lined by a single layer of immunopositive flattened endothelial cells with a vascular lumen, in the presence or absence of lymphocytes and absence of erythrocytes [20]. LMVD in tumor sections was determined by averaging the number of total lymphatic vessels in all the fields of each slide, including within the tumor or at the periphery of the tumor. The mean visual microvessel density was calculated as the average of four counts (two of the authors: two microscopic fields). Discordant cases were recounted, and the consensus resolved any discrepancy of more than 10% of the microvessel count.

ECM1 IHC staining results were expressed in two ways [10]: (1) the percentage of cells staining on a graduated percentage (0-100%); 10-30% of tumor cells in the section were positive (+); 30-60% of tumor cells were positive (++); 60-100% of tumor cells were positive (+++). For analysis as a dichotomous variable, staining < 10% was classified as ECM1-negative and ≥ 10% classified as ECM1-positive; this allowed comparisons to be made against previous studies. (2) The percentage of positive staining = (the numbers of positive samples/the numbers of samples tested) × 100%. The semi-quantitative assessment of VEGF-C staining was conducted as described for the ECM1 staining assessment.

### Statistics

Data were analyzed using SPSS (version 17.0; Chicago, IL, USA). The distribution of quantitative variables was tested for normality using the Kolmogorov-Smirnov test. Data which were normally distributed were applied to parametric statistical analysis and results expressed as the mean ± the standard error of the mean (S.E.M.). Unpaired Student's *t*-test was used to compare two sets of data and a one-way analysis of variance (ANOVA) with Dunnett's post-test was used for comparisons of more than two data sets. Non-parametric statistics were applied to data that was not normally distributed. *χ*^2 ^tests, Yates' correction, or Fisher's exact test were used for qualitative independent variables. The Bonfferoni correction of the α-value for multiple comparisons was conducted. Correlations between two variables were assessed using Spearman's rho test. All statistical tests were two-sided. A *P*-value of less than 0.05 was considered significant.

## Results

### LMVD assessment

D2-40 is a commercially available mouse monoclonal antibody directed against human podoplanin, which is a mucin-type transmembrane protein present in lymphatic endothelial cells [21]. It is a specific marker for lymphatic endothelium and has proven valuable in distinguishing lymph vessels from blood vessels and detecting lymphatic invasion in various malignant neoplasms [22-24]. It has been reported that the basal epithelial cell layers of the epidermis and the myoepithelial cells of human breast tissue, prostate and salivary gland tissue can also be stained by D2-40; but the morphology of these cells are distinct from the characteristic morphology of lymphatic endothelium [25,26].

**Figure 1:**
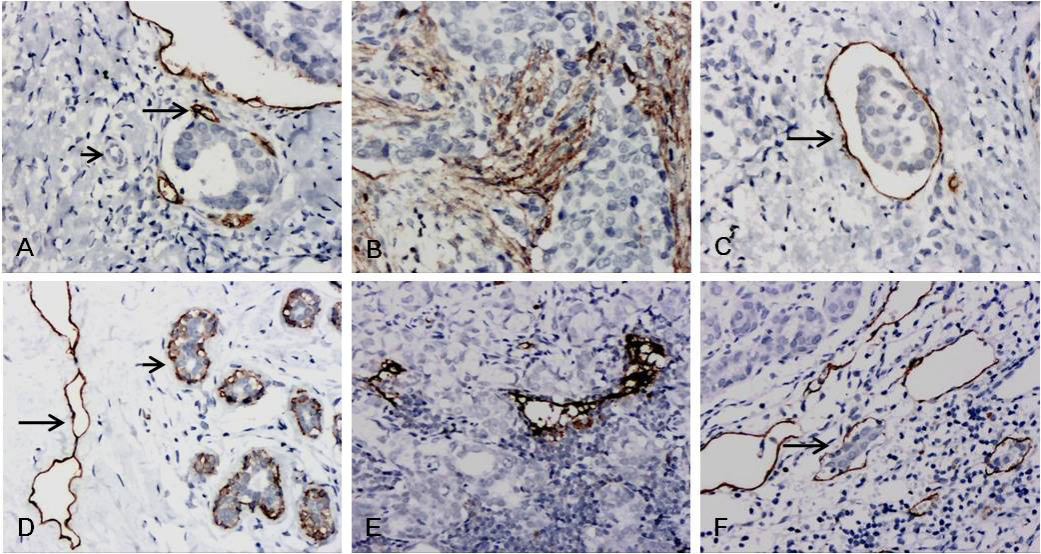
**D2-40 labelled LMVD with IHC staining (200×)**. A-C Invasive ductal breast cancer: A. Stained lymphatic microvessels within a peripheral tumor (long arrow) with dilated tubes. Capillary vessel (short arrow) and tumor cell clusters were not stained; B. The central-tumor vascular structures appear linear, small and flattened, cluttered and densely arrayed; C. a cluster of tumor cells (arrow) in a stained vessel, or "tumor emboli". D. Luminal epithelial cells with D2-40 negative staining. Myoepithelial cells from normal ducts and lobules had positive D2-40 immunostaining (short arrow), but with a granular, branching membranous staining pattern that is distinct from the characteristic staining pattern of lymphatic endothelium (long arrow). E-F Metastatic lymph nodes: E. stained lymphatic microvessels in the metastasis, with irregular lumens and cracked tube walls; F. dilated lymphatic microvessel sub-capsular metastasis with no staining of tumor cells within it; defined as lymphatic vessel invasion (LVI) (arrow).

In the present study, D2-40 staining was mainly located in the cytoplasm or cell membrane of the lymphatic endothelial cells, whilst the tumor cells and blood vessel endothelium had no D2-40 staining. The ductal cancer in situ (DCIS) foci displayed weak residual discontinuous myoepithelial staining. We found that the tumor lymphatic vessel invasion (LVI) was established when at least one tumor cell cluster ("tumor emboli") was clearly visible inside a D2-40 positive lymph vessel, according to criteria established by Hasebe et al. [27]. Representative examples of the staining obtained in these studies are shown in Figure 1.

For LMVD assessment, we found that the mean number of visual microvessels was 12.95 ± 1.73 (range 1.04-42.10) lymphatic microvessels per 200× field (LMV per 200× field) in the 41 breast cancer specimens. Within the same subjects, the mean LMVD in normal breast tissue and lymph nodes were 2.24 ± 0.18 (range 1.00-5.03) and 5.49 ± 0.52 (range 0-32.01) LMV per 200× field, respectively. The differences observed in the LMVD between the three tissue types were statistically significant (Friedman test, *P *< 0.01). Dunnett's post-test showed that the mean LMVD in tumor tissues was higher than that observed in the normal breast tissues (*P *< 0.01) and lymph nodes (*P *< 0.01). However, LMVD differences between the normal tissue and the lymph nodes were not statistically significant (*P *> 0.05). The mean LMVD in the lymph nodes of the metastasis group was higher than that in the non-metastasis group (Mann-Whitney test, *P *= 0.003). Differences in the LMVD in the metastasis and non-metastasis groups was not found to be statistically significant in the cancer tissues, nor the normal breast epithelium (*P *= 0.409 and *P *= 0.377, respectively) (Table 1).

**Table 1:** Comparison of LMVD between the metastasis and non-metastasis groups

	LMVD (LMV per 200× field of vision)			
			
Tissue type	Metastasis group	Non-metastasis group	U	*P*
Cancer tissues	15.16 ± 2.94	11.05 ± 1.97	177.0	0.409
Normal tissues	2.42 ± 0.27	2.09 ± 0.24	176.0	0.377
Lymph nodes	9.32 ± 2.16	2.18 ± 0.32	97.5	0.003*

* *P *< 0.05 is considered statistically significant

Abbreviation: *LMVD *lymphatic microvessel density; *LMV *per 200× field of vision: lymphatic microvessel 200× field of vision

Our data show that LMVD in the breast cancer tissue was significantly higher than that in the normal tissues. We also found that LMVD in the lymph nodes with metastasis was higher than that without metastasis. Lymphatic vessels have discontinuous basement membranes and lack tight interendothelial junctions. Hence, it is possible that lymphatic vessels might be easier for tumor cells to enter than blood vessels [2], and that LMVD enhancement could significantly increase the potential for tumor cells to invade the surface of lymphatic vessels [28].

### *ECM1 *and *VEGF-C *mRNA and protein expression

We used real-time RT-PCR to determine the mean relative expression levels of *ECM1 *mRNA (Table 2) and *VEGF-C *mRNA (Table 3) in breast cancer specimens, normal epithelia and lymph nodes from the patients. Differences in the *ECM1 *mRNA expression levels among these tissues were statistically significant (one-way ANOVA, *P *< 0.01) (Table 2). Multiple comparison analysis (Tukey's test) showed that *ECM1 *mRNA expression levels in the breast cancer samples were significantly higher overall, compared to the normal tissue (*P *< 0.05) or to the lymph nodes (*P *< 0.05); however, no differences were found between normal tissues and lymph nodes (*P *> 0.05). In general, the results of *VEGF-C *mRNA expression among the three tissue types showed the same trends as those obtained for *ECM1 *(Table 3).

**Table 2:** *ECM1 *expression in breast cancer specimens, normal epithelium and lymph nodes

		*ECM1*mRNA expression		Positive	Staining grades of ECM1				
						
Tissue type	N		F	rate (%)	-	+	++	+++	***χ***^**2**^
Cancer tissues	41	1.25 ± 0.33^A^		75.6^D^	10	22	7	2	
Normal tissues	41	0.46 ± 0.10^B^		9.8^E^	37	3	1	0	
Lymph nodes	41	0.38 ± 0.18^**C**^	4.86**	31.7^**F**^	28	9	3	1	39.08**

** *P *< 0.01, *P *< 0.05 is considered statistically significant

"+", "++" and "+++" for ECM1 immunochemistry staining was all grouped together as "+"

A vs. B and A vs. C: *P *< 0.05, respectively; B vs. C: *P *> 0.05

*χ*^2 ^division:α = 0.05/4 = 0.0125; D vs. E and D vs. F: *P *< 0.001, respectively; E vs. F: *P *> 0.0125

**Table 3:** *VEGF-C *expression in breast cancer specimens, normal epithelium and lymph nodes

		*VEGF-C *mRNA expression		Positive	Staining grades of VEGF-C				
						
Tissue type	N		F	rate (%)	-	+	++	+++	***χ***^**2**^
Cancer tissues	41	2.63 ± 0.32^A^		80.5^D^	8	20	9	4	
Normal tissues	41	1.08 ± 0.17^B^		46.3^E^	22	17	2	0	
Lymph nodes	41	1.33 ± 0.17^C^	15.05***	36.6^F^	26	10	3	2	23.08***

*** *P *< 0.001, *P *< 0.05 is considered statistically significant

"+", "++" and "+++" for VEGF-C immunochemistry staining was all grouped together as "+"

A vs. B and A vs. C: *P *< 0.01, respectively; B vs. C: *P *> 0.05

*χ*^2 ^division:α = 0.05/4 = 0.0125; D vs. E and D vs. F: *P *< 0.01, respectively; E vs. F: *P *> 0.0125

**Figure 2:**
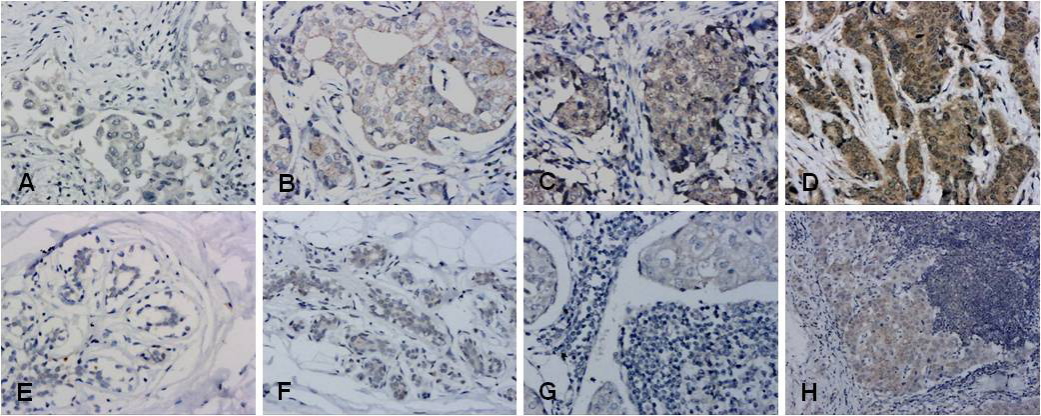
**Representative IHC staining of ECM1**. A-D: ECM1 was detected primarily in the cytoplasm of breast cancer cells (200×): A. ECM1 negative; B. + ECM1 staining; C. ++ ECM1 staining; D. +++ ECM1 staining. E-F normal breast epithelium (200×): E. ECM1 negative; F. breast ductal epithelial cells with cytoplasmic ECM1 staining classified as +. G-H lymph node metastases: G. metastatic cells with ECM1 staining (200×) classified as +; H. cytoplasm of metastatic cells with ECM1 staining (100×) classified as ++.

We found that ECM1 was mainly located in the cytoplasm of the cells, with scant staining noted on the cell membrane or the stroma; no nuclear staining was seen. Within the draining lymph nodes, ECM1 staining was specific for the metastatic cancer cells and occurred primarily in their cytoplasm. Notably, in the normal breast epithelium, there was little or no staining at all. Representative examples of the ECM1 staining patterns are shown in Figure 2. The ECM1 positive staining rates among tumor tissue (31, 75.6%), normal breast tissue (4, 9.8%) and lymph nodes (13, 31.7%) were significantly different (*χ*^2 ^= 39.08, *P *< 0.01). Multiple comparison (*χ*^2 ^division, α = 0.0125) analysis showed the ECM1 positive staining rate in cancer tissue was higher than in normal tissue and the lymph nodes (*P *< 0.001). Differences in the ECM1 positive staining rate between normal tissues and lymph nodes were not significant, however (*P *> 0.0125, Table 2).

**Figure 3:**
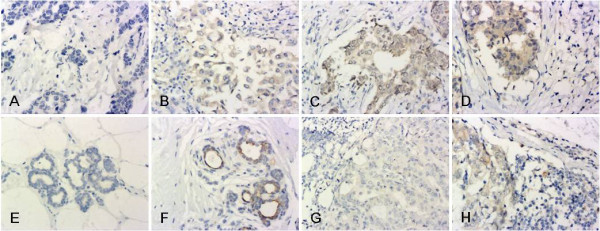
**Representative IHC staining of VEGF-C (200×)**. A-D: VEGF-C staining was mainly located in the cytoplasm of breast cancer cells: A. VEGF-C negative; B. + VEGF-C staining; C. ++ VEGF-C staining; D. +++ VEGF-C staining. E-F normal breast epithelium: E. VEGF-C negative; F. breast ductal epithelial cells classified ++ for cytoplasmic VEGF-C staining. G-H lymph node metastases: G. metastatic cells were negative for VEGF-C staining; H. cytoplasm of metastatic cells classified + for VEGF-C staining.

In breast cancer cells, VEGF-C staining was observed in the cytoplasm; such staining was often more intense at the invasive edge or in the intraductal component (Figure 3). In contrast, very little or no staining was observed in normal ductal epithelium. According to the criteria used to evaluate the immunostaining, VEGF-C expression in the cancer specimens (33/41, 80.5%) was higher than that in the normal tissues and lymph nodes (19/41, 46.3% and 15/41, 36.6%, respectively; *P *< 0.01). However, the difference in the values obtained from the normal epithelium and the matched lymph nodes was not significant (*P *> 0.0125, Table 3).

### Differences in *ECM1 *and *VEGF-C *expression between the metastatic and non-metastatic groups

Statistically significant differences in *ECM1 *mRNA expression in tumor tissues between the metastasis group and the non-metastasis group were not found (Mann-Whitney test, *P *= 0.314); no statistically significant differences were found for the normal breast epithelium tissues or the lymph nodes, between the metastasis and non- metastasis groups (*P *= 0.754 and *P *= 0.178, respectively; Table 4). The results of *VEGF-C *mRNA expression between the metastasis group and the non-metastasis group showed similar trends as those observed for *ECM1 *(Table 5).

**Table 4:** Comparison of *ECM1 *expression levels between the metastasis- and non-metastasis groups

	*ECM1 *mRNA expression			ECM1 positive rate (%)		
				
Tissue type	Metastasis	Non-metastasis	*P*	Metastasis	Non-metastasis	*P*
Cancer tissues	1.11 ± 0.39	1.48 ± 0.54	0.314	78.9(15/19)	72.7(16/22)	> 0.05
Normal tissues	0.53 ± 0.18	0.40 ± 0.09	0.754	15.8(3/19)	4.55(1/22)	> 0.05
Lymph nodes	0.62 ± 0.36	0.20 ± 0.13	0.178	68.4(13/19)	(0/22)	< 0.01*

* *P *< 0.05 is considered statistically significant

Within parenthesis: number of eligible cases

**Table 5:** Comparison of *VEGF-C *expression levels between the metastasis- and non-metastasis groups

	*VEGF-C *mRNA expression			VEGF-C positive rate (%)		
				
Tissue type	Metastasis	Non-metastasis	*P*	Metastasis	Non-metastasis	*P*
Cancer tissues	2.27 ± 0.39	2.92 ± 0.48	0.455	68.4 (13/19)	90.9 (20/22)	> 0.05
Normal tissues	0.95 ± 0.18	1.18 ± 0.27	0.948	47.4 (9/19)	45.5 (10/22)	> 0.05
Lymph nodes	1.21 ± 0.23	1.46 ± 0.24	0.314	68.4 (13/19)	9.1 (2/22)	< 0.001*

* *P *< 0.05 is considered statistically significant

Within parenthesis: number of eligible cases

Differences in the ECM1 positive staining rates between metastatic (15/19, 78.9%) and non-metastatic (16/22, 72.7%) tumors were not significant (*P *> 0.05). Similar results were obtained for the normal tissue groups (metastatic: 3/19, 15.8%; non-metastatic:1/22, 4.55%) (Fisher's exact test, *P *> 0.05, Table 4). The ECM1 positive rate in the lymph node metastases was 68.4% (13/19). Likewise, no differences were apparent in VEGF-C expression in the three tissues (i.e., cancer tissue, normal tissue and lymph nodes) between the metastasis and non-metastasis groups (Fisher's exact test, *P *> 0.05, Table 5).

In the metastatic group, the difference in the ECM1 positive staining rate between the primary tumor (15/19, 78.9%) and the metastatic focus (13/19, 68.4%) was not statistically significant (*P *> 0.05, Table 4). In two of the cases, we found that the primary tumor was ECM1 negative, whilst ECM1 was expressed in the corresponding lymph node metastases. Similarly, the difference in the VEGF-C positive staining rate between the primary tumor and the metastatic focus was not significant (*P *> 0.05, Table 5). In addition, the VEGF-C staining rates in the two tissue types (i.e. cancer tissues and lymph nodes) were both 68.4% (13/19), although the cases that had positive staining did not all coincide with each other.

### *ECM1 *and *VEGF-C *expression profiles and clinical characteristics

We evaluated whether correlations exist between expression of *ECM1 *or *VEGF-C *and the clinicopathological characteristics of the disease, (i.e. age; histological type or grade; tumor size; lymph node, ER, and PR status and Her-2/neu score). Tables 6 and 7, summarize the *ECM1 *and *VEGF-C *data, respectively. *ECM1 *mRNA expression was not associated with any of the clinicopathological characteristics tested (Unpaired *t *test with Welch's correction, *P *> 0.05). The ECM1 protein positive rate was found to be associated with the status of the ER (*χ*^2^, *P *= 0.045). The ER status of patients with ECM1-positive tumors were more likely to be positive than those without ECM1 staining. However, ECM1 staining was not correlated with tumor size, lymph node status, PR status or the Her-2/neu score (Fisher's exact test, *P *> 0.05). We found that *VEGF-C *mRNA and protein expression were not associated with any of the clinicopathological characteristics tested (*P *> 0.05, respectively; Table 7).

**Table 6:** Correlations between *ECM1 *expression and clinicopathological characteristics

Clinicopathological characteristics	N	ECM1 positive		*P*	*ECM1 *mRNA expression	*P*
					
		N	rate (%)			
Age (years)						
< 60	30	22	73.3		1.54 ± 0.43	
≥ 60	11	9	81.8	0.700	0.68 ± 0.46	0.183
Histological grade (invasive ductal)						
I+II	16	12	75.0		1.76 ± 0.71	
III+IV	20	16	80.0	0.720	1.18 ± 0.39	0.483
Tumor size						
T1	15	10	66.7		1.49 ± 0.60	
T2	23	19	82.6		1.34 ± 0.46	
T3	3	2	66.7	0.512	0.11 ± 0.08	0.275
Lymph nodal status						
N0	22	16	72.7		1.48 ± 0.54	
N1	12	10	83.3		1.32 ± 0.58	
N2	5	3	60.0		0.81 ± 0.60	
N3	2	2	100	0.121	0.54 ± 0.50	0.424
Estrogen receptor status						
-	10	5	50.0		1.02 ± 0.62	
+~+++	31	26	83.9	0.045*	1.40 ± 0.41	0.611
Progesterone receptor status						
-	8	4	50.0		1.32 ± 0.90	
+~+++	33	27	81.8	0.082	1.30 ± 0.37	0.987
HER2/neu score						
- (0-1)	13	11	84.6		1.48 ± 0.69	
+ (2-3)	28	20	71.4	0.458	1.23 ± 0.39	0.757

"+", "++" and "+++" for ECM1 immunochemistry staining was all grouped together as "+"

* *P *< 0.05 is considered statistically significant

**Table 7:** Correlations between *VEGF-C *expression and clinicopathological characteristics

Clinicopathological characteristics	N	VEGF-C positive		*P*	*VEGF-C *mRNA expression level	*P*
					
		N	rate (%)			
Age (years)						
<60	30	23	76.7		2.41 ± 0.33	
≥ 60	11	10	90.9	0.412	3.20 ± 0.76	0.505
Histological grade (invasive ductal)						
I+II	16	14	87.5		2.72 ± 0.58	
III+IV	20	14	70.0	0.257	2.65 ± 0.45	0.908
Tumor size						
T1	15	12	80.0		3.00 ± 0.63	
T2	23	18	78.3		2.14 ± 0.31	
T3	3	3	100	0.670	4.58 ± 1.68	0.237
Lymph nodal status						
N0	22	20	90.9		2.92 ± 0.48	
N1	12	8	66.7		2.10 ± 0.53	
N2	5	3	60.0		2.51 ± 0.86	
N3	2	2	100	0.606	2.65 ± 0.07	0.710
Estrogen receptor status						
-	10	9	90.0		2.42 ± 0.63	
+~+++	31	24	77.4	0.653	2.69 ± 0.38	0.606
Progesterone receptor status						
-	8	6	75.0		2.14 ± 0.75	
+~+++	33	27	81.8	0.642	2.75 ± 0.35	0.217
HER2/neu score						
- (0-1)	13	10	76.9		2.70 ± 0.66	
+ (2-3)	28	23	82.1	0.693	2.60 ± 0.36	0.918

"+", "++" and "+++" for VEGF-C immunochemistry staining was all grouped together as "+"

### Correlations between LMVD and ECM1 or VEGF-C

**Figure 4:**
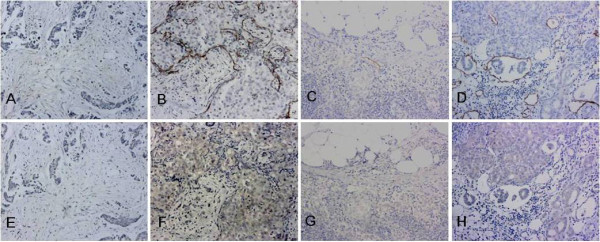
**Representative examples of D2-40 and ECM1 staining in the matched sections (100×)**. Sections with ECM1-positive staining had higher lymphatic microvessel densities. A-D: Lymphatic microvessels labelled with D2-40. E-H: ECM1 IHC staining: Panels E and G: ECM1 negative; Panels F and H: ++ ECM1 staining. A-B, E-F: invasive ductal breast cancer; C-D, G-H: lymph nodes with metastasis. Panels A and E, B and F, C and G, D and H represent matched sections obtained from the same specimen, respectively.

We found that histological sections that were ECM1-positive had higher LMVDs (Figure 4). As shown in Table 8, differences in LMVD between the ECM1-positive cases and ECM1-negative cases were statistically significant for both the tumor tissues (Mann-Whitney test, *P *= 0.045), and the lymph nodes (Mann-Whitney test, *P *< 0.001). A positive correlation was further established between the LMVD and ECM1 staining intensity in both the breast cancer sections, and the lymph node sections (Spearman's correlation coefficient 0.347 and 0.604, respectively; *P *< 0.05, Table 9). The correlation was not linear, however.

**Table 8:** Correlation between ECM1 staining and LMVD (LMV per 200× field of vision)

	ECM1 staining			
			
Tissue type	-	+	U	*P*
Cancer tissues	7.50 ± 1.63(10)	15.17 ± 2.14(31)	85.50	0.045*
Lymph nodes	2.71 ± 0.62(28)	11.46 ± 2.77(13)	48.00	0.000***

"+", "++" and "+++" for ECM1 immunochemistry staining was all grouped together as "+".

* *P *< 0.05 and *** *P *< 0.001 is considered statistically significant

Within parenthesis: number of eligible cases. Abbreviation: LMVD: lymphatic microvessel density; LMV per 200× field of vision: lymphatic microvessel 200× field of vision

**Table 9:** Correlation between ECM1 staining intensity and LMVD (LMV per 200× field of vision)

	ECM1 expression intensity					
			
Tissue type	-	+	++	+++	r	*P*
Cancer tissues	7.50 ± 1.63(10)	14.73 ± 2.81(22)	15.57 ± 3.80(7)	1.50 ± 2.50(2)	0.347	0.026*
Lymph nodes	2.71 ± 0.62(28)	10.44 ± 3.06(9)	14.00 ± 9.08(3)	13(1)	0.604	< 0.001*

* *P *< 0.05 is considered statistically significant

Within parenthesis: number of eligible cases. Abbreviation: LMVD: lymphatic microvessel density; LMV per 200× field of vision: lymphatic microvessel 200× field of vision

We noted that the LMVD in the VEGF-C-positive lymph nodes was higher than that in the VEGF-C-negative ones (Mann-Whitney test, *P *< 0.001); LMVD in the lymph nodes was correlated with VEGF-C staining (Spearman's correlation coefficient: 0.566, *P *< 0.001). However, LMVD in the breast cancer specimens was not associated with VEGF-C staining (*P *> 0.05, Table 10).

**Table 10:** Correlation between VEGF-C staining and LMVD (LMV per 200× field of vision)

	VEGF-C staining			
			
Tissue type	-	+	U	*P*
Cancer tissues	8.88 ± 2.57(8)	13.94 ± 2.04(33)	93.50	0.211
Lymph nodes	3.15 ± 1.19(26)	11.88 ± 2.32(25)	75.00	< 0.001***

"+", "++" and "+++" for VEGF-C immunochemistry staining was all grouped together as "+"

*** *P *< 0.05 is considered statistically significant

Within parenthesis: number of eligible cases. Abbreviation: LMVD: lymphatic microvessel density; LMV per 200× field of vision: lymphatic microvessel 200× field of vision

**Figure 5:**
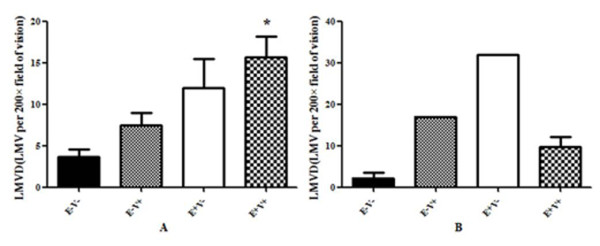
**LVMD in breast cancer specimens and lymph nodes with ECM1 and VEGF-C staining**. A: LMVD in both ECM1- and VEGF-C-positive (E+V+) tumor specimens was higher than in both ECM1- and VEGF-C-negative (E-V-) ones (*P *< 0.05). B: The LMVD within the 41 lymph nodes from different assemblies (i.e. E-V-, E-V+, E+V- and E+V+) for both ECM1 and VEGF-C staining were statistically different (*P *< 0.05), but no statistically significant tendency was identified between the assemblies. Abbreviations: E-V-: negative for both ECM1 and VEGF-C staining; E-V+: negative for ECM1 staining but positive for VEGF-C staining; E+V-: positive for ECM1 staining but negative for VEGF-C staining; E+V+: positive for both ECM1 and VEGF-C staining.

We further analysed the LMVD in breast cancer tissue and lymph nodes for both ECM1 and VEGF-C staining (Table 11). The LMVD in both ECM1- and VEGF-C-positive (E+V+) tumor specimens was higher than that in both the ECM1- and VEGF-C-negative (E-V-) ones (Mann-Whitney test, *P *= 0.029; Figure 5A). Additionally, the LMVD in both E+V- and E-V+ tumor specimens was higher than in the E-V- specimens (Mann-Whitney test, *P *> 0.05). We also found that LMVD in E+V+ tumor specimens was higher than in the E-V+ and E+V- ones, although the differences did not reach statistical significance (Mann-Whitney test, *P *> 0.05). However, LMVD in the lymph nodes of the different assemblies for both ECM1 and VEGF-C staining (i.e. E-V-, E-V+, E+V- and E+V+) showed a statistically significant difference (one way ANOVA, *P *= 0.025), but did not show a significant tendency (Figure 5B).

**Table 11:** LMVD with ECM1 and VEGF-C staining (LMV per 200× field of vision)

ECM1 staining	VEGF-C staining(Cancer tissues)			ECM1 staining	VEGF-C staining(Lymph nodes)		
					
	-	+	*P*		-	+	*P*
**-**	3.67 ± 0.88(3)	7.43 ± 1.59(7)		-	2.20 ± 1.24(5)	17.0(1)	
**+**	12.0 ± 3.44(5)	15.7 ± 2.45(26)	0.054	+	32.0(1)	9.75 ± 2.37(12)	0.025*

"+", "++" and "+++" for ECM1 or VEGF-C immunochemistry staining was all grouped together as "+"

* *P *< 0.05 is considered statistically significant

Within parenthesis: number of eligible cases. Abbreviation: LMVD: lymphatic microvessel density; LMV per 200× field of vision: lymphatic microvessel 200× field of vision

## Discussion

The lymphangiogenic properties of ECM1 have been reported elsewhere [6]. However, whether this protein is involved in the formation of new lymphatic vessels in tumor progression is not fully understood. The present study highlighted a positive correlation between ECM1 protein expression and LMVD, in both the tumor specimens and the lymph nodes. In addition, VEGF-C expression in the lymph nodes was correlated with LMVD. These results indicate that ECM1 has a closer relationship with LMVD than VEGF-C. LMVD reflects the status of lymphangiogenesis and the incidence of lymphatic metastasis. Therefore, like VEGF-C, ECM1 appears to be a potent enhancer of tumor lymphangiogenesis and may contribute to an increased rate of metastatic spread of breast cancer cells to the lymph nodes. Furthermore, the LMVD in both ECM1- and VEGF-C-positive tumor specimens was statistically higher than that noted in the both of the ECM1- and VEGF-C-negative ones as well as the ECM1- or VEGF-C-positive ones; this suggests that ECM1 and VEGF-C might have a synergistic effect on lymphangiogenesis in breast cancer.

In the present study, cytoplasmic ECM1 was significantly elevated in breast cancer specimens, compared to the peritumoral normal counterparts from the same patients. Han et al. [29] and Wang et al. [10] also reported that ECM1 was overexpressed in breast cancer tissues. Moreover, the *ECM1 *mRNA and protein levels detected by real-time RT-PCR and IHC were consistent with each other, suggesting that the elevated ECM1 protein expression levels may derive from increased transcription of the gene.

A previous study demonstrated that breast cancers with lymph node metastasis were more likely to be ECM1-positive (10/13, 76.9%) than those without metastasis (3/9, 33.3%) [10]. However, we failed to identify any statistically significant differences in ECM1-staining from different patients with regard to lymph node metastasis; this finding is in accordance with an analysis of a single hospital-based cohort of patients, in which ECM1 expression was not statistically associated with the status of lymph node metastasis [8]. Differences observed between our findings and those reported by Wang et al. [10] and Lal et al. [8] could be related to a number of differences between the studies; for example, differences in the number of cases (i.e., sample size effects), use of antibodies from different suppliers, or the different compositions of the histological types. Participants in the Lal et al. [8] study, as well as our own, mainly presented with infiltrating breast cancer, but Wang et al. [10] did not specify the breast cancer stage. In our study, differences in the relative *ECM1 *mRNA expression levels between the metastasis and non-metastasis cases were not statistically significant. Additionally, we found *VEGF-C *expression had no statistically significant difference between the metastasis and non-metastasis cases, which is consistent with that reported by Kinoshita et al. [30]. However, other studies have reported associations between VEGF-C expression and lymph node metastasis [31,32]. Methodological variability, particularly with respect to the antibodies and specimens used, may cause inconsistencies in the results obtained in the different studies. Recent studies have demonstrated that the expansion of lymphatic networks within the lymph nodes occurred prior to the onset of metastasis [3]. Therefore, it appears likely that the status of lymph node metastasis cannot be used as an early predictor of lymphatic invasion. These findings, as well as the absence of a correlation between ECM1 and VEGF-C expression in the tumor cells and lymph node metastasis may be explained by the fact that metastatic establishment in lymph nodes is a complex process in which multiple growth factors are involved. Lymphangiogenesis initiated by lymphangiogenic factors secreted from tumor cells occurs at the onset of metastasis. Hence, our finding that ECM1 and VEGF-C expression is not associated with lymph node metastasis is plausible. One possible explanation is that ECM1 or VEGF-C might be responsible for the early events relating to lymphatic spread prior to lymph node metastasis [30].

To explore whether ECM1 expression was associated with the metastatic character of the breast cancer cells, we analyzed the ECM1 expression profiles between the primary tumors and the metastatic foci in individuals with metastasis. Theoretically, the metastatic focus in the nodes and the primary nests are consanguine. Additionally, we found two cases that were ECM1-negative in the primary tumor, but ECM1-positive in the corresponding lymph node metastases. Han et al. [29] and Wang et al. [10] also found a case that was ECM1-negative in the primary tumor but exhibited ECM1-positive staining in its corresponding metastatic focus. This suggests that ECM1 is associated with breast cancer cells that have a potential for metastasis, although the exact mechanism remains unclear. Recently, it has been reported that ECM1 is selectively expressed in Type 2 helper T cells (T_H_2 lymphocytes), and regulates T_H_2 cell migration via expression of KLF2 and S1P_1 _[33]. Whether ECM1 regulates metastasis associated genes, or interacts with other extracellular matrix proteins, or both, or is involved in tumor cells migration is currently unknown.

Recently published work suggests that overexpression of TFAP2α or TFAP2γ induced *ECM1 *expression in human mammary epithelial cells could result in modified ER responsiveness [34]. In our study, ER positive tumors appeared more likely to be ECM1-positive. This finding indicates that ECM1 is associated with estrogen responsiveness in breast cancer. Further investigations are needed to prove this as well as the role of ECM1 in the breast estrogen-receptor-axle. It will be interesting to see whether ECM1 could become a new target for breast cancer hormonotherapy.

It is possible that ECM1 alone is not sufficient to facilitate lymphangiogenesis, which may require multiple lymphangiogenic factors. VEGF-C is the most extensively studied molecule for tumor lymphangiogenesis and we found that VEGF-C has potential synergy with ECM1 for facilitating lymphatic metastasis. One limitation of this study was the relatively small sample size. Nevertheless, our findings support a potential role for ECM1 in the lymphatic progression of breast cancer, an area that will require further study to explore the mechanisms involved.

## Conclusions

Our data demonstrate that both *ECM1 *and *VEGF-C *mRNA and protein were overexpressed in breast cancer specimens compared to their corresponding normal counterparts and axillary lymph nodes. ECM1 protein expression was positively correlated with estrogen responsiveness and LMVD, but was not correlated with the status of the lymph node metastasis in this study. ECM1 and VEGF-C may have a synergistic effect on lymphangiogenesis to facilitate the lymphatic metastasis of breast cancer.

## Competing interests

The authors declare that they have no competing interests.

## Authors' contributions

QWW and ZMZ designed and participated in all stages of the study. QWW and HQS performed most of the experiments. JL participated in analysis of the IHC data, in the statistical analyses and discussion of the results. HQS and YFH conducted modified radical mastectomy for breast cancer and patient follow ups. QMY and QLY participated in the IHC staining experiments. All authors read and approved the final manuscript.

## Pre-publication history

The pre-publication history for this paper can be accessed here:

http://www.biomedcentral.com/1471-2407/12/47/prepub
